# Performance Characterization of a Switchable Acoustic Resolution and Optical Resolution Photoacoustic Microscopy System

**DOI:** 10.3390/s17020357

**Published:** 2017-02-12

**Authors:** Mohesh Moothanchery, Manojit Pramanik

**Affiliations:** School of Chemical and Biomedical Engineering, Nanyang Technological University, 62 Nanyang Drive, Singapore 637459, Singapore; mmohesh@ntu.edu.sg

**Keywords:** photoacoustic imaging, AR-PAM, OR-PAM, microscopy, deep tissue imaging

## Abstract

Photoacoustic microscopy (PAM) is a scalable bioimaging modality; one can choose low acoustic resolution with deep penetration depth or high optical resolution with shallow imaging depth. High spatial resolution and deep penetration depth is rather difficult to achieve using a single system. Here we report a switchable acoustic resolution and optical resolution photoacoustic microscopy (AR-OR-PAM) system in a single imaging system capable of both high resolution and low resolution on the same sample. Lateral resolution of 4.2 µm (with ~1.4 mm imaging depth) and lateral resolution of 45 μm (with ~7.6 mm imaging depth) was successfully demonstrated using a switchable system. In vivo blood vasculature imaging was also performed for its biological application.

## 1. Introduction

Photoacoustic microscopy (PAM) is an emerging hybrid in vivo imaging modality, combining optics and ultrasound, which can provide penetration beyond the optical diffusion limit with high resolution. This approach can provide deeper imaging than other optical modalities and has been successfully applied to in vivo structural, functional, molecular, and cell imaging [1,2,3,4,5,6,7,8,9]. PAM overcomes the limitations of other existing optical modalities combining optical contrast with ultrasound resolution. In PAM, the contrast is related to the optical properties of the tissue, but the resolution is not limited by optical diffusion due to multiple photon scattering. Unlike optical coherence tomography (OCT), PAM does not rely on ballistic or backscattered light. Any light, including both singly and multiply scattered photons, contributes to the imaging signal. As a result, the imaging depth in PAM is relatively large. The key advantages of PAM include (1) combination of high optical contrast and high ultrasonic resolution; (2) good imaging depth; (3) no speckle artifacts; (4) scalable resolution and imaging depth with the ultrasonic frequency; (5) use of non-ionizing radiation (both laser and ultrasound pose no known hazards to humans); and (6) relatively inexpensive.

In PAM, a short laser pulse irradiates the tissue/sample. Due to absorption of light by the tissue chromophores (such as melanin, hemoglobin, and water), there is a temperature rise, which in turn produces pressure waves emitted in the form of acoustics waves. A wideband ultrasonic transducer receives the acoustic signal (known as photoacoustic (PA) waves) outside the tissue/sample boundary. In acoustic resolution photoacoustic microscopy (AR-PAM) deep tissue imaging can be achieved with weak optical and tight acoustic focusing [10,11,12]. Since AR-PAM lateral resolution is dependent on the ultrasound focus, one can achieve high lateral resolution (~45 µm with 50 MHz focused ultrasound transducer with numerical aperture (NA) 0.44) with an imaging depth of up to 3 mm, as the PA signal in AR-PAM does not depend on the ballistic photons. Resolving single capillaries acoustically need ultrasonic transducers greater than 400 MHz central frequency; however, at this frequency the penetration depth will be less than 100 µm. In optical resolution photoacoustic microscopy (OR-PAM), the lateral resolution can be improved by a tight optical focus; one can achieve a lateral resolution of up to 0.5 µm in the reflection mode and up to 0.2 µm lateral resolution in the transmission mode [13,14,15,16,17,18,19,20]. There were other techniques employed to attain super resolution imaging capability including nonlinear enhancement [17,21], use of double excitation process [22], and use of a photonic nanojet [23,24]. OR-PAM can clearly resolve single capillaries or even a single cell [25]. However, the penetration depth is rather limited due to light focusing and it can image up to ~1.2 mm inside the biological tissue [19]. Therefore, in summary AR-PAM can image deeper, but with lower resolution and OR-PAM can image with very high resolution but limited imaging depth. The imaging speed of the AR- and OR-PAM system mainly depends on the pulse repetition rate of the laser source [26].

Not many efforts have been put to integrate both these systems together. Mostly, two different imaging scanners are used for imaging. However, hybrid imaging with both optical and acoustic resolution PAM enables imaging with scalable resolution and depth. In one approach, the optical and ultrasound focus have been shifted for doing both AR- and OR-PAM. However, since the light focus and ultrasound focus are not aligned, the image quality and resolution was not optimal [27]. In another approach, an optical fiber bundle was used to deliver light for OR- and AR-PAM [28]. In this approach, they have used two separate lasers (high energy laser at 532 nm for the AR and a low energy high repetition rate laser at 570 nm for the OR), making the system inconvenient, expensive, and not suitable for applications including oxygen saturation measurements [29]. In any of these techniques, AR-PAM was not having dark field illumination and hence there were strong photoacoustic signals from the tissue surface. The use of dark field illumination can reduce the generation of strong photoacoustic signals from the skin surface hence deep tissue imaging can be performed using ring-shaped illumination as the detection sensitivity of deep photoacoustic signals will be higher compared to brightfield illumination [12]. Here, we report a switchable AR- and OR-PAM (AR-OR-PAM) imaging system capable of both high-resolution imaging as well as low resolution deep tissue imaging on the same sample utilizing dark field illumination. We use the same laser for both systems. The AR-OR-PAM system was characterized in terms of spatial resolution and imaging depth using phantom experiments. In vivo blood vasculature imaging was performed on mouse ear for demonstrating its biological application.

## 2. System Description 

### 2.1. The Switchable Acoustic Resolution-Optical Resolution-Photoacoustic Microscopy (AR-OR-PAM) System

**Figure 1:**
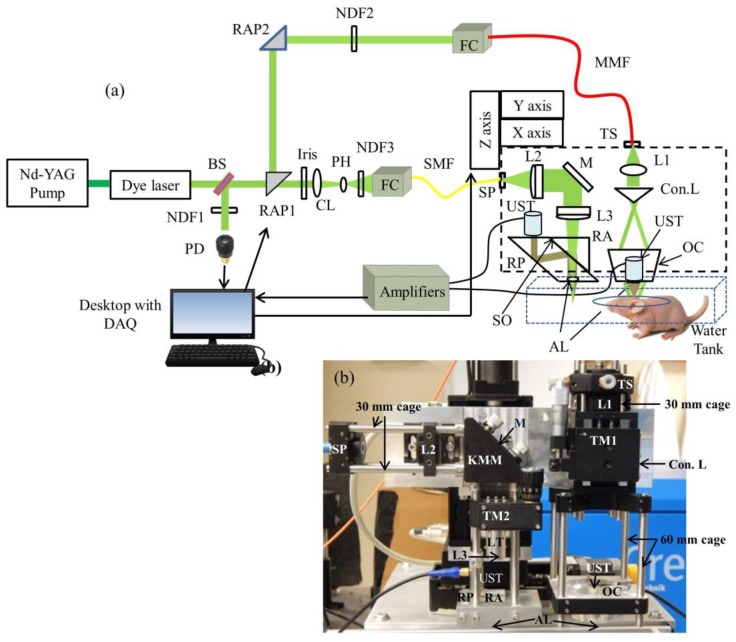
(**a**) Schematic of the Acoustic Resolution—Optical Resolution—Photoacoustic Microscopy (AR-OR-PAM) imaging system. BS: beam sampler; NDF: neutral density filter; RAP: right angle prism; PD: photodiode; CL: condenser lens; PH: pinhole; FC: fiber coupler; UST: ultrasound transducer; MMF: multimode fiber; SMF: single mode fiber; DAQ: data acquisition card; TS: translation stage; Con.L: conical lens; L1: convex lens; L2 & L3: achromatic lens; RA: right angle prism; RP: rhomboid prism; OC: optical condenser; M: mirror; SP: slip plate; LT: lens tube; TM: translation mount; KMM: kinematic mirror mount; AL: acoustic lens; (**b**) Photograph of the prototype AR-OR-PAM system.

The schematic of the AR-OR-PAM system is shown in Figure 1a. Figure 1b shows the photograph of the switchable AR-OR-PAM scanning head. This AR-OR-PAM system employs a nanosecond tunable laser system, consisting of a diode-pumped solid-state Nd-YAG laser (INNOSLAB, Edgewave, Wurselen, Germany) and a dye laser (Credo-DYE-N, Sirah dye laser, Spectra Physics, Santa Clara, CA, USA). The laser system was tunable between 559–576 nm using Rhodamine 6G dye. The wavelength range can be changed depending on the dye used. For example, using DCM dye, the wavelength range can be changed to 602–660 nm. For AR-PAM scanning, the laser beam was diverted using a right angle prism, RAP1 (PS915H-A, Thorlabs, Newton, NJ, USA), placed on a computer controlled motorized stage (CR1/M-Z7, Thorlabs). The diverted beam passed through another right angle prism, RAP2 (PS915H-A, Tholabs), and a variable neutral density filter, NDF2 (NDC-50C-4M, Thorlabs), and coupled on to a multimode fiber, MMF (M29L01, Thorlabs) using a combination of objective (M-10X, Newport, Irvine, CA, USA) and XY translator (CXY1, Thorlabs), which acts as the fiber coupler (FC). The fiber out was fixed on the stage using a translator (TS) (CXY1, Thorlabs). The beam out from the fiber passed through a collimating lens, L1 (LA1951, Thorlabs), and then passed through a conical lens (Con.L), having an apex angle of 130° (1-APX-2-B254, Altechna, Vilnius, Lithuania) to provide a ring-shaped beam. The conical lens was placed on a translating mount, TM1 (CT1, Thorlabs). The ring shaped beam was allowed to focus weakly onto the subject with the focal region coaxially overlapping the ultrasonic focus inside the tissue using a homemade optical condenser (OC) (cone angles: 70°, 110°), having a 50 MHz ultrasonic transducer (UST) (V214-BB-RM, Olympus-NDT, Waltham, MA, USA) in the center. An acoustic lens (AL) (LC4573, Thorlabs) having a radius of curvature of 4.6 mm and a 6 mm diameter was attached using a UV curing optical adhesive (NOA61, Thorlabs) to the bottom of the transducer, which provided an acoustic focal diameter of ~46 µm. In an optically clear medium, the optical focus was around 2 mm in diameter, which was wider than the ultrasonic focus. This type of dark field illumination is beneficial for deep tissue imaging where there are no strong signals from the tissue surface. The laser repetition rate (LRR) was set to be 1 kHz, and the laser energy at focus can be varied up to 30 µJ per pulse. The optical illumination on the object surface was donut shaped with a dark center so that no strong photoacoustic signals were produced from the surface on the object within the ultrasonic field of view. In our setup, all components were integrated and assembled in an optical cage setup. For AR, both 30 mm and 60 mm optical cages (OC connected in 60 mm cage) were used. The use of the cage system made the AR setup compact and easier to assemble and align.

For the OR-PAM setup, the rotational stage (holding the RAP1) would rotate at 90° so that the laser beam went straight and was reshaped by an iris (ID12/M, Thorlabs) and then focused by a condenser lens, CL (LA4327, Thorlabs), and passed through a 50 μm pinhole, PH (P50S, Thorlabs), for spatial filtering. The filtered beam was attenuated by a variable neutral density filter, NDF3 (NDC-50C-4M, Thorlabs), and launched on to a single-mode fiber, SMF (P1-460B-FC-1, Thorlabs), using a single mode fiber coupler, FC (F-91-C1, Newport). The output port of the single-mode fiber was placed on a slip plate positioner, SP (SPT1, Thorlabs). The output beam from the SMF was then collimated by an achromatic lens, L2 (32-317, Edmund Optics, Barrington, New Jersey, United States) was reflected by a stationary elliptical mirror, M (PFE10-P01, Thorlabs), was fixed on a Kinematic mirror mount, KMM (KCB1, Thorlabs), and filled the back aperture of another identical achromatic lens, L3, placed on a translation mount, TM2 (SM1Z, Thorlabs). The achromatic lens was placed on the translation mount with the help of a lens tube, LT (SM05L10, Thorlabs). The effective clear aperture of the achromatic lens through the tube was 10.9 mm, which makes the effective numerical aperture (NA) of the achromatic lens as 0.11. The beam then passed through an optoacoustic beam combiner consisting of a right angled prism, RA (PS615, Thorlabs) and a rhomboid prism, RP (47-214, Edmund optics) with a layer of silicon oil, SO (DMPS1M, Sigma Aldrich, St. Louis, MI, USA) in between. The silicon oil layer acts as optically transparent and acoustically reflective film. An acoustic lens, AL (LC4573, Thorlabs) provided acoustic focusing (a focal diameter of ~46 μm), was attached at the bottom of the rhomboid prism. The ultrasonic transducer, a 50 MHz center frequency (V214-BB-RM, Olympus-NDT), was placed on top of the rhomboid with an epoxy layer from a single part of a two part epoxy (G14250, Thorlabs) for effective coupling. To maximize the detection sensitivity, the optical and acoustic foci were aligned confocally. The laser repetition rate for the OR-PAM was set to 5 kHz and the laser energy at focus could be varied up to 200 nJ per pulse. Like AR, the OR systems components were also integrated and assembled in a 30 mm optical cage system.

The AR-OR combined system was attached to a homemade plate that helps in switching between AR and OR scanhead easily by sliding the scanhead on top of the imaging area. At present, the *y-*axis translation stage used has a range of 5 cm; therefore, the switching between the AR and OR systems was done by manual sliding. However, if one uses the *y-*axis translation stage with a 10 cm range, manual transition can be avoided. The AR-OR combined scanner head was attached to a 3-axis motorized stage (PLS 85 for *X* and *Y* axis, VT 80 for *Z* axis, PI—Physik Instrumente, Karlsruhe, Germany). All three stages were controlled by a 3-axis controller (SMC corvus eco, PI micos) connected to the computer. For photoacoustic imaging, the bottom of the AR-OR-PAM scanner head was submerged in a water-filled tank (13 cm × 30 cm) for acoustic coupling. An imaging window of 7 cm × 7 cm was opened at the bottom of the tank and sealed with a polyethylene membrane for optical and acoustic transmission. The PA signal acquired by the UST was amplified by two amplifiers (ZFL-500LN, Mini Circuits, Brooklyn, NY, USA) each having a 24 dB gain, and was recorded using a data acquisition (DAQ) card, (M4i.4420, Spectrum, Grosshansdorf, Germany) in a desktop computer (Intel xeon E5-1630 3.7 GHz processor, 16 GB RAM, 64 bit windows 10 operating system). The DAQ card had a 16 bit analog-to-digital converter (ADC), a 250 Ms/s sampling rate, 2 channels, and a 4 GB on-board memory. The same desktop computer was used for both AR and OR-PAM systems. The scanning and data acquisition was controlled using Labview software (National Instrument). Two-dimensional continuous raster scanning of the imaging head was used during image acquisition. The time-resolved PA signals were multiplied by the speed of sound, 1540 m/s in soft tissue [30] to obtain an A-line. Multiple A-lines were captured during the continuous motion of the Y stage to produce a two-dimensional B-scan. Multiple B-scans of the imaging area were captured and stored in the computer. MATLAB (MathWorks, Natick, MA, USA) was used to process and obtain the maximum amplitude projection (MAP) photoacoustic images. 

The synchronization of the data acquisition and the stage motion was controlled by the signal from a photodiode (PD) (SM05PD1A, Thorlabs). A beamsampler, BS (BF10-A, Thorlabs), was placed in front of the laser beam diverted a small portion of the beam (5%) to the PD. A neutral density filter, NDF 1 (NDC-50C-4M, Thorlabs), was placed in front of the PD to control the energy falling on the PD. The PD signal can also used for compensating pulse-to-pulse laser energy variations during data acquisition. All experiments were done at a laser wavelength of 570 nm in this work.

### 2.2. Laser Safety

For in vivo imaging, the maximum permissible pulse energy is governed by American National Standards Institute (ANSI) laser safety standards [31]. The safety limit varies with illumination wavelength, pulse duration, exposure duration, and exposure area. The maximum pulse energy by a single laser pulse ($MPE_{SP}$) on the skin surface should not exceed $MPE_{SP}=\;2C_{A}10^{-2}\;J/{\mathrm{cm}}^{2}$, where $C_{A}$ the wavelength correction factor, is unity for visible wavelength range (400–700 nm). The irradiance should not exceed 200 mW/cm^2^ if a point on skin is exposed to more than 10 s. In the case of raster scanning, a point on the skin will not be exposed for 10 s; hence, the maximum permissible exposure ($MPE_{AVE}$) is limited by $1.1\;C_{A}t^{0.25}\;\mathrm{mJ}/{\mathrm{cm}}^{2}$, where t denotes the exposure duration in seconds.

For AR-PAM, the diameter of the optical focus at the ultrasound focus was 2 mm. Having a minimum pixel separation of 15 µm, an average of 133 (N) adjacent laser pulses overlap at the ultrasound focus. At 1 kHz LRR, the exposure time was 133 ms, so the maximum pulse energy for the pulse train ($MPE_{TRAIN}$) was 664 mJ/cm^2^ ($1.1\;C_{A}t^{0.25}$). The $MPE_{SP}\;$for the pulse train was $MPE_{AVG}=MPE_{TRAIN}/N\;=\;664/133\;=\;5\;\;\mathrm{mJ}/{\mathrm{cm}}^{2}$. The current AR-PAM system can deliver per pulse energy of 0.32 mJ/cm^2^ (30 µJ/pulse, 2 mm diameter focus), which is well below the $MPE_{SP}$ safety limit. For AR-PAM experiments, we used a pulse energy of 30 µJ/pulse for imaging depth and 6 µJ/pulse for the resolution test and in vivo ear blood vasculature imaging.

For OR-PAM, we believe the effect of optical aberrations at the prism surface and acoustic lens might have reduced the objective NA from 0.11 to 0.075, which will give a spot size diameter of 3.9 µm (which agrees with our lateral resolution). Assuming the optical focus is 150 micron below the skin surface for in vivo imaging, the surface spot size was 22.5 µm in diameter. Having a minimum pixel separation of 2 µm, an average of 11 (N) adjacent laser pulses overlaps on the skin surface. At 5 kHz LRR, the exposure time was 2.4 ms. Therefore, the $MPE_{TRAIN}$ was 238 mJ/cm^2^. The $MPE_{SP}$ for the pulse train was $MPE_{AVG}=\;MPE_{TRAIN}/N\;=\;238/11\;=\;21.6\;\mathrm{mJ}/{\mathrm{cm}}^{2}$. The current OR-PAM system can deliver an $MPE_{SP}$ of 20.4 mJ/cm^2^ (90 nJ/pulse, 0.075 NA) at the skin surface (close to the safety limit). For OR-PAM experiments, we used a pulse energy of 20 nJ/pulse for the resolution test and 90 nJ/pulse for imaging depth and in vivo ear blood vasculature imaging.

## 3. Experimental Methods

In order to evaluate the system performance of the switchable AR-OR-PAM system, a series of experiments were conducted to determine the spatial resolution and the maximum imaging depths for both AR- and OR-PAM. In vivo imaging was also done using the switchable system to show the biological imaging capability of the system.

### 3.1. Spatial Resolution Quantification

The lateral resolution of the AR and OR system was determined by imaging a 100 nm gold nanoparticle (742031, Sigma Aldrich). To determine the resolution of the AR-PAM system, a single nanoparticle was scanned with a step size of 5 microns. Similarly, the nanoparticle was scanned with a step size of 0.5 microns in order to find the resolution of the OR-PAM system. The photoacoustic amplitude along the central lateral direction of the nanoparticle image was fitted to a Gaussian function. The full width at half maximum (FWHM) of the Gaussian fit was considered the lateral resolution. Theoretically, the optical diffraction-limited lateral resolution for the OR-PAM was calculated from 0.51 λ/NA, where λ was the laser wavelength, and NA was the numerical aperture of the objective. Similarly, the theoretical lateral resolution for the AR-PAM was determined using the equation 0.72 λ/NA, where λ was the central acoustic wavelength, and NA was the numerical aperture of the ultrasonic transducer. The photoacoustic axial spread profile from the nanoparticle was used to determine the axial resolution of the system. Both OR-PAM and AR-PAM share the same axial resolution since the same ultrasound transducer (and the focusing lens) was used in both systems. The axial resolution was determined by acoustic parameters according to 0.88 c/Δf, where c is the speed of sound in soft tissue, and Δf is the frequency bandwidth of the ultrasonic transducer. Since the size of the nanoparticle was much smaller than the axial resolution, the axial spread profile can be considered as axial point spread function of the imaging system. The FWHM of the envelope gives the axial resolution. The axial resolution was also calculated by numerically shifting and summing two A-line signals and by checking whether the two peaks could be differentiated in the envelope with a contrast-to-noise ratio (CNR) greater than 2. The CNR was plotted against the shift between the two impulse responses. The contrast was defined as the difference between the smaller of the two peaks in the photoacoustic envelope and the valley between the peaks. The noise was the standard deviation in the background photoacoustic signal.

### 3.2. USAF Resolution Test Target Imaging

The lateral resolution of the AR and OR system was further validated imaging a USAF 1951 test target (R1DS1P, Thorlabs). Initially, a 5 mm × 5 mm area (Group numbers 2 to 7) were scanned using AR-PAM. The scan step size was 10 µm in both X and Y directions. Similarly, a 1.3 mm × 1.3 mm area (Group numbers 4 to 7) was scanned using OR-PAM with a step size of 0.5 µm in both *X* and *Y* directions. Finally, a 0.3 mm × 0.3 mm area consisting of the smallest groups (Group Numbers 6 and 7) were scanned using OR-PAM imaging with a step size of 0.5 µm in both *X* and *Y* directions.

### 3.3. Imaging Depth

To determine the maximum imaging depth of both AR-PAM and OR-PAM, a black tape was inserted obliquely on a chicken tissue. A single B-scan image was captured using both AR-PAM and OR-PAM. The signal-to-noise ratio (SNR) was also determined at the maximum imaging depth. SNR is defined as V/n, where V is the peak-to-peak PA signal amplitude, and n is the standard deviation of the background noise.

### 3.4. In Vivo Imaging of Mouse Ear Blood Vasculature

To demonstrate in vivo imaging using the combined system, the ears of 4-week-old female mice with body weights of 25 g, procured from InVivos Pte. Ltd. (Singapore), were used. Animal experiments were performed according to the approved guidelines and regulations by the institutional Animal Care and Use committee of Nanyang Technological University, Singapore (Animal Protocol Number ARF-SBS/NIE-A0263). The animal was anesthetized using a cocktail of ketamine (120 mg/kg) and xylazine (16 mg/kg) injected intraperitoneally (dosage of 0.1 mL/10 g, body weight). After removing hair from the ear, the mouse was positioned on a platform that also has a miniature plate to position the ear. The animal was further anesthetized with vaporized isoflurane system (1 L/min oxygen and 0.75% isoflurane) during the imaging period. The imaging region was placed into contact with the polyethylene membrane using ultrasound gel. Using AR-PAM, a large area (9 mm × 7 mm) of the ear was first imaged, using a step size of 15 µm in the *Y* direction and 30 µm in the *X* direction. The same area (4.5 mm × 5 mm) was scanned using OR-PAM with a step size of 2 µm in the *Y* direction and 3 µm in the *X* direction.

## 4. Results and Discussion

### 4.1. Spatial Resolution of the Imaging System

**Figure 2:**
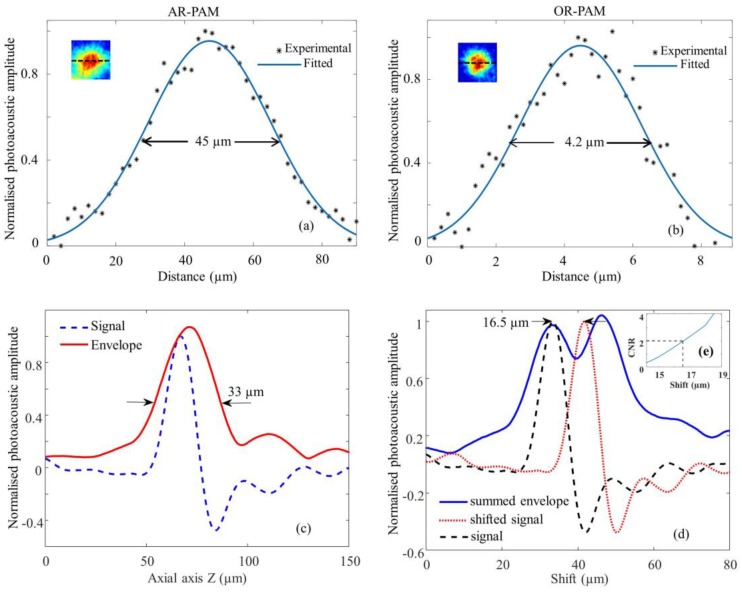
Spatial resolution test of the AR-OR-PAM system: Lateral resolution estimated by imaging gold nanoparticles ~100 nm diameter, Black (*) dots: photoacoustic signal; blue line: Gaussian-fitted curve; (**a**) AR-PAM; (**b**) OR-PAM. The inset shows the representative AR-PAM image in (**a**) and OR-PAM image in (**b**) of the single gold nanoparticle; (**c**) Photoacoustic axial spread profile and its envelope; (**d**) Simulated photoacoustic shift-and-sum A-line signals. The dashed line and dotted line indicate two photoacoustic signals 16.5 µm apart. The solid line indicates the summed envelope of the two shifted signals; (**e**) Contrast-to-noise ratio (CNR) versus the shift distance between the two signals.

The lateral resolution of the AR-PAM is shown in Figure 2a. The measured lateral resolution is 45 µm determined by FWHM. Similarly, lateral resolution of OR-PAM is shown in Figure 2b. The measured lateral resolution determined from the FWHM is 4.2 µm. The inset of the figures shows the corresponding PAM image of the gold nanoparticle.

Figure 2c shows the axial spread profile of the averaged PA signal from the gold nanoparticle and its envelope. The axial resolution was measured to be 33 µm. The experimentally determined axial resolution matches closely to the theoretical axial resolution of 29 µm. The simulated results in Figure 2d show that we can distinguish the two absorbers separated by 16.5 µm with a CNR of 2. Figure 2e shows the plot of CNR versus axial shift.

### 4.2. USAF Resolution Test Target Imaging

**Figure 3:**
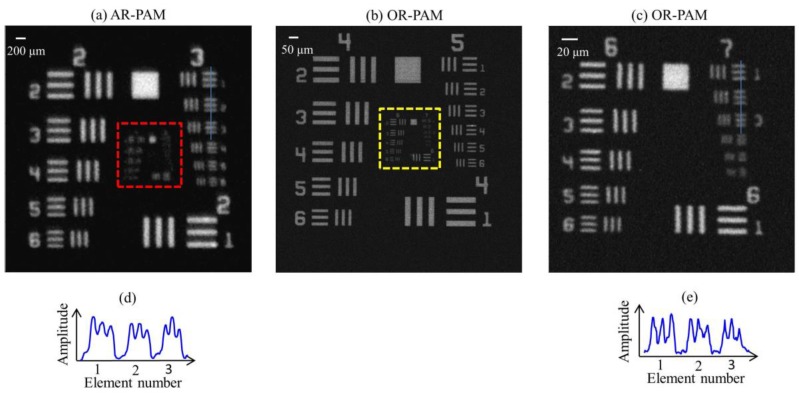
Lateral resolution test of the AR-OR-PAM system: (**a**) AR-PAM image of an air force resolution test target; (**b**) OR-PAM image of the red dotted area; (**c**) OR-PAM image of the yellow dotted region of the test target; (**d**) The cross-sectional profile of the first two elements in Group 3 of the resolution target, blue line in (**a**); (**e**) The cross-sectional profile of the first three elements in Group 7 of the resolution target, blue line in (**c**).

MAP AR-PAM image of a USAF resolution test target is shown in Figure 3a. From Figure 3a,d, we can see that the AR-PAM system is capable of resolving 49.61 µm line pairs (Group 3, Element 3) with a modulation transfer function (MTF) of 0.28. Figure 3b is a MAP OR-PAM image done on the red dotted area shown in Figure 3a.

Figure 3c shows the MAP OR-PAM image done on the yellow dotted area on Figure 3b. From Figure 3c,d, we can see that the OR-PAM system can clearly resolve 3.91 µm line pairs (Group 7, Element 1) with an MTF of 0.64. Theoretically, the optical diffraction-limited lateral resolution for the OR-PAM is 2.6 µm. The experimentally measured lateral resolution was poorer than the diffraction-limit estimate, which might be due to wavefront aberrations. Similarly, the theoretical lateral resolution for the AR-PAM is 46 µm. The theoretical resolution agrees well with our experimental data.

### 4.3. Imaging Depth

**Figure 4:**
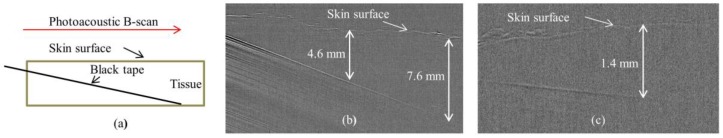
Single B-scan PA image of a black tape inserted obliquely in a chicken tissue. (**a**) Schematic diagram; (**b**) AR-PAM image; (**c**) OR-PAM image.

Figure 4a shows the schematic of a black tape obliquely inserted on chicken tissue. Figure 4b shows the B-scan PA image from AR-PAM. It is evident that the AR-PAM system can clearly image the black tape down to ~7.6 mm beneath the tissue surface. Similarly, using the OR-PAM system, we can clearly image the black tape down to ~1.4 mm beneath the tissue surface. For AR-PAM, the SNR at 4.6 mm and 7.6 mm imaging depth were 2.5 and 1.4, respectively. In case of OR-PAM, the SNR of the target object (black tape) at 1.4 mm imaging depth was 1.5.

### 4.4. In Vivo Imaging of Mouse Ear Blood Vasculature

**Figure 5:**
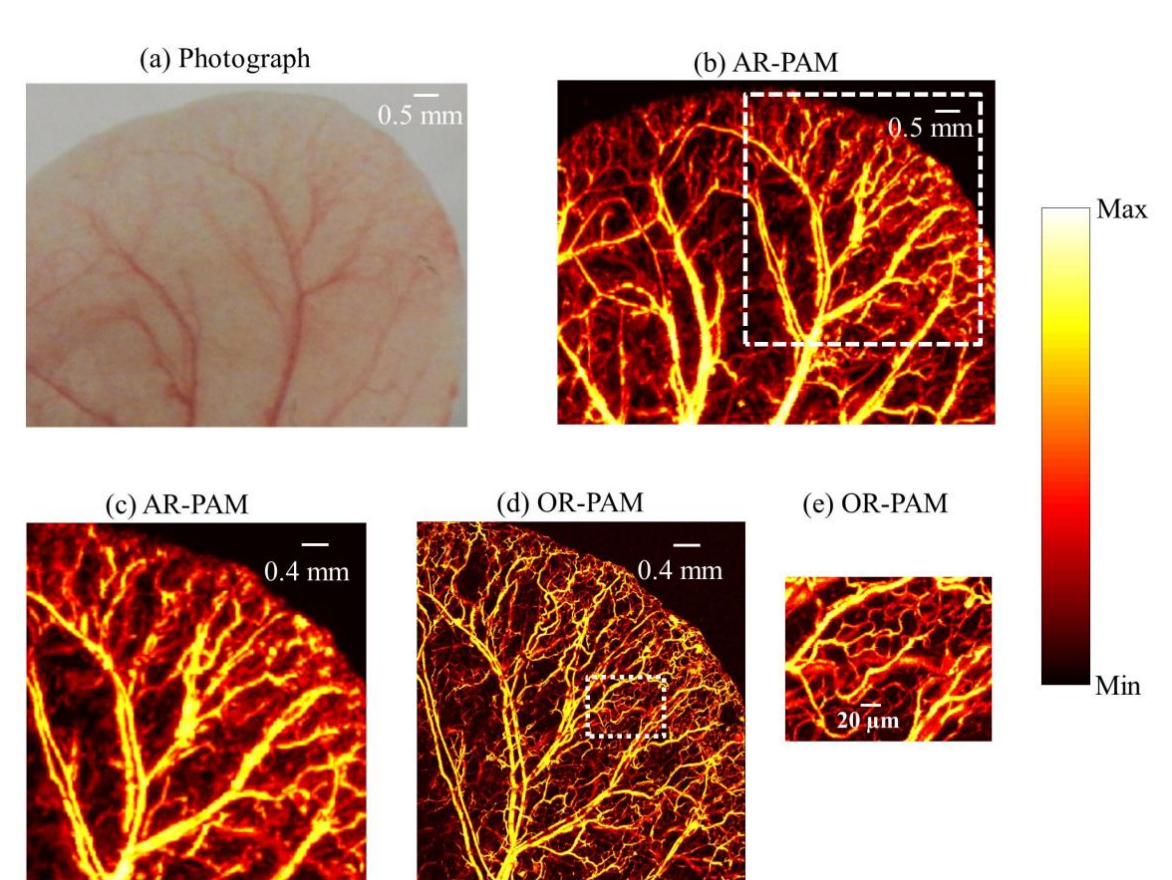
In vivo photoacoustic image of mouse ear: (**a**) photograph of the mouse ear vasculature; (**b**) AR-PAM image; (**c**) close up of the region of interest (ROI) in (**b**) as shown by white dash line; (**d**) OR-PAM image of the same ROI; (**e**) close up of the region of interest in (**d**) as shown by white dotted line.

Figure 5a shows the photograph of the mouse ear vasculature. A unidirectional B-scan imaging of 9 mm × 7 mm area using AR-PAM took 10 min to complete. The MAP image of AR-PAM is show in Figure 5b. Figure 5c shows the zoomed out image of the white dotted region in Figure 5b. The same area as in Figure 5b (4.5 mm × 5 mm) was scanned using OR-PAM (imaging time 50 min). The MAP image of the OR-PAM is shown in Figure 5d. Figure 5c,d are the same region scanned with AR-PAM and OR-PAM. We can see OR-PAM can clearly resolve single capillaries that AR-PAM cannot resolve. AR-PAM can resolve deep vessels thicker than 45 µm. Figure 5e shows the zoomed out area (white dotted region in Figure 5d). Due to the high resolution of the OR-PAM, the region appears clearer, and smaller structures are also visible.

In summary, a switchable AR-OR-PAM system that can achieve high-resolution imaging utilizing optical focusing as well as deep tissue imaging using dark field illumination and acoustic focusing was developed. This combined photoacoustic microscopy system can provide high spatial resolution, which makes the system important for applications including imaging of angiogenesis and drug response, where imaging single capillaries as well as deep vasculatures will be important. Further improvement in the system can be done by replacing the switchable plate with a 10 cm traveling motorized stage (*y-*axis). Wavefront aberration corrections for the OR-PAM will improve the lateral resolution further. Delivering higher pulse energy to the AR-PAM will improve the SNR and imaging depths as well. The limitations of the proposed technique include the scanning speed. Currently longer scanning time is required, which can be further reduced by acquiring data in both directions during imaging. High speed imaging using OR-PAM was reported by the use of a high repetition rate laser and a water immersible MEMS (microelectromechanical system) mirror [32]. Simultaneous image acquisition using both AR-PAM and OR-PAM is not possible at the moment. Developing a system that can do simultaneous data acquisition using OR-PAM and dark field AR-PAM would have been more advantageous.

## 5. Conclusions

A switchable acoustic resolution and optical resolution photoacoustic microscopy system that can achieve both high-resolution imaging at lower imaging depth and lower resolution imaging at higher imaging depth was developed. This is the first combined system using the same laser, which can be easily switched between OR-PAM and dark field AR-PAM. The combined system will have a 4.2 µm resolution with a 1.4 mm imaging depth, as well as a 45 µm resolution with a 7.6 mm imaging depth. The system is made of minimal homemade components, making it easier to assemble, align, and build. Using the combined system, in vivo imaging was successfully demonstrated. The developed system can be used for pre-clinical imaging. Major preclinical applications include imaging of angiogenesis, microcirculation, tumor microenvironments, drug response, brain functions, biomarkers, and gene activities.
